# Development and Application of a Blocking ELISA for the Detection of Feline Calicivirus Antibodies Based on Monoclonal Antibodies Against VP1 Protein

**DOI:** 10.1155/tbed/7831655

**Published:** 2026-06-29

**Authors:** Haojie Wang, Lihong Xue, Bowen Shan, Shuyan Wu, Tongqin An, Changqing Yu, Changyou Xia, He Zhang

**Affiliations:** ^1^ State Key Laboratory of Animal Disease Control and Prevention, Harbin Veterinary Research Institute, Chinese Academy of Agricultural Sciences, Harbin, China, caas.cn; ^2^ School of Advanced Agricultural Sciences, Yibin Vocational and Technical College, Yibin, Sichuan, China

**Keywords:** antibody detection, blocking ELISA, feline calicivirus, monoclonal antibody, vaccine evaluation, VP1 protein

## Abstract

In this study, nine monoclonal antibodies (mAbs) against the FCV VP1 protein were successfully prepared using traditional hybridoma technology, and all of them could simultaneously recognize linear and conformational epitopes. Their subtypes mainly included IgG1/kappa, IgG2a/kappa, IgG1/lambda, IgG2b/kappa, IgG2b/lambda, and IgM/kappa. Based on the mAb 8F9 with the highest blocking activity, a blocking enzyme‐linked immunosorbent assay (ELISA) method for detecting cat calicivirus antibodies was established. After systematic optimization, the antigen coating concentration was determined to be 1.0 μg/mL, the serum dilution was 1:8, and the dilution of horseradish peroxidase (HRP)‐labeled mAb was 1:400. The critical value for detecting cat serum was 23.5%, the sensitivity was 94.9%, and the specificity was 93.02%. There was no cross‐reaction with common pathogens such as feline parvovirus and feline mycoplasma. The repeatability was good, with intra‐batch and inter‐batch coefficients of variation (CV) <10%. Clinical validation showed that the total coincidence rate with the indirect ELISA method recommended in the local standard was 96.19% (kappa = 0.9). The sensitive and specific blocking ELISA method established in this study can provide reliable technical support for FCV antibody screening, vaccine immune evaluation, and specific pathogen‐free (SPF) cat breeding.

## 1. Introduction

Cats are one of the important companion animals for humans. According to the 2026 China Pet Industry White Paper (consumer report), the market size of cat consumption in China reached 152 billion yuan in 2025, and the number of urban cats reached 72.89 million. Moreover, the number advantage is still increasing. However, diseases are an important factor threatening the cats. Among them, feline upper respiratory tract infection syndrome accounted for 6.7% (from the 2026 China Pet Industry White Paper), and feline calicivirus (FCV) is one of the important pathogens causing symptoms of feline upper respiratory tract diseases. It is reported that in the survey of 11 cat pathogen infection rates in 14 provinces of China from 2022 to 2023, the positive rate of FCV reached 11.56%, ranking among the top three pathogens with the highest positive rates [1]. FCV belongs to the genus *Vesivirus* within the family Caliciviridae [2, 3]. Since its first identification in the United States in 1957, FCV has spread worldwide, causing substantial health burdens in cat populations [4]. Beyond its health consequences, FCV also represents a substantial economic threat worldwide. Direct costs include veterinary consultations, diagnostic testing, hospitalization, and supportive treatments, while indirect costs arise from reduced breeding efficiency, outbreak management in multicat environments, and welfare‑related losses. Although FCV does not infect humans, the rapid growth of the global companion animal market means that morbidity and mortality in cats can translate into significant economic repercussions for owners, the pet care industry, and animal welfare organizations. Clinical manifestations of FCV infection include fever, conjunctivitis, rhinitis, oral ulceration, and chronic gingivostomatitis; less frequently, dermatological lesions, lameness, and pneumonia may also develop. Infections with highly virulent systemic strains (VS‐FCV) can induce widespread cytolysis and systemic vasculitis, often leading to rapid fatal outcomes [5, 6]. The FCV capsid consists primarily of the VP1 protein, which contains both conserved and variable regions, determining viral antigenicity and host immune recognition. Currently, available diagnostic methods for FCV—including virus isolation, RT‑polymerase chain reaction (PCR), and serological assays—suffer from limitations such as lengthy procedures, requirements for specialized equipment, or insufficient sensitivity and specificity, highlighting the need for improved detection tools.

FCV is a nonenveloped RNA virus with a genome of ~7.7 kb. Its icosahedral nucleocapsid exhibits characteristic cup‐shaped depressions on the surface. The viral genome contains three open reading frames (ORFs): ORF1 encodes a polyprotein of about 1800 amino acids that undergoes proteolytic cleavage to facilitate viral replication; ORF2 encodes the major capsid protein VP1; and ORF3 encodes the minor capsid protein VP2 [7, 8]. VP1 is structurally organized into six domains (A–F), among which domains A, B, D, and F are relatively conserved, whereas domains C and E are highly variable [9, 10]. Domain E specifically interacts with the feline junctional adhesion molecule‐1 (fJAM‐1) receptor on host cells, mediating viral entry. Domain F, located at the C‐terminus, is highly conserved and can bind nonneutralizing antibodies [11]. Importantly, domains C, D, and E contain critical neutralizing epitopes that elicit host neutralizing antibody responses, making VP1 the key antigenic target for developing FCV vaccines and diagnostic assays [12, 13].

Accurate diagnosis is essential for effective disease prevention and control [14]. Currently available detection methods for FCV include virus neutralization tests, nucleic acid assays, and antigen‐ or antibody‐based detection [15–17]. While the virus neutralization test is considered the gold standard, it is labor‐intensive, time‐consuming, and reliant on cell culture, limiting its utility for large‐scale sample screening. PCR and quantitative real‐time PCR offer high throughput, sensitivity, and specificity [18, 19] but require expensive instrumentation and specialized technical expertise. Isothermal amplification techniques such as loop‐mediated isothermal amplification (LAMP) and recombinase polymerase amplification (RPA) provide rapid, equipment‐free alternatives for nucleic acid detection; however, they are not suitable for applications such as vaccine immunogenicity evaluation [20]. For antigen detection, immunochromatographic test strips are widely used for point‐of‐care diagnosis in companion animals, though their sensitivity remains suboptimal. Among serological methods, enzyme‐linked immunosorbent assay (ELISA) is commonly employed to assess vaccine‐induced immune responses. Indirect ELISA is cost‐effective and straightforward but may suffer from reduced specificity due to serum heterogeneity [21, 22]. In contrast, blocking ELISA offers notable advantages: using coated viral antigen or recombinant protein, it employs monoclonal antibodies (mAbs) to competitively inhibit serum antibody binding, thereby providing a specific and efficient measure of the antibody presence and titer [23]. This format has been widely adopted for the serological monitoring of viral infections. Nevertheless, reports on mAb‐based ELISA for FCV remain limited. The most recent study, by Yuan et al. [24]. in 2014, described a double‐antibody sandwich ELISA using FCV‐specific mAbs. However, this method does not offer the same rapidity as immunochromatographic strips for field or clinical use in companion animal settings.

As the use of FCV vaccines becomes increasingly common, the need for a reliable antibody detection method has grown more pressing. Such a method is essential for assessing vaccine immunogenicity, identifying seronegative animals, and supporting the development of specific pathogen‐free (SPF) cat colonies. To address this need, this study utilized the conventional hybridoma technique to generate nine mAbs against the FCV VP1 protein. After systematically evaluating their blocking activity, the most potent mAb was selected. Using this antibody, a blocking ELISA was developed that demonstrated high stability, specificity, sensitivity, and efficiency. This established assay is intended to serve as a robust technical tool for screening SPF cat populations, detecting antibody‐negative individuals, and evaluating the immune response induced by FCV vaccines.

## 2. Materials and Methods

### 2.1. Animal Ethics Statement

Female BALB/c mice (aged 4–6 weeks) were obtained from Beijing Weitonglihua Laboratory Animal Technology Co., Ltd. All animal procedures were carried out in an Animal Biosafety Level 2 (ABSL‐2) facility at the Harbin Veterinary Research Institute, Chinese Academy of Agricultural Sciences, in accordance with institutional guidelines for animal care and use. The experimental protocol was reviewed and approved by the Harbin Veterinary Research Institute Ethics Committee (Number: 240723‐01‐GR).

### 2.2. Cells, Viruses, Plasmid, and Serum

Feline kidney cells and SP2/0 myeloma cells were cultured in Dulbecco’s Modified Eagle Medium (DMEM, Cat: D6429, Sigma–Aldrich, USA) supplemented with 10% fetal bovine serum (FBS, Cat: F4135, Sigma–Aldrich, USA) at 37°C in a 5% CO2 atmosphere. FCV strain and the plasmid pMAL‐C5x (+) were maintained in our laboratory. Positive serum samples against common feline pathogens, including FCV, feline herpesvirus type I, feline panleukopenia virus, *Salmonella*, *Escherichia coli*, and *Mycoplasma*, were also preserved in our laboratory.

### 2.3. Expression and Purification of Recombinant FCV VP1 Protein

Viral RNA was extracted from FCV using a commercial viral RNA extraction kit (Cat: D9424, TIANGEN, China) and reverse‐transcribed into cDNA with the HiScript IV 1st Strand cDNA Synthesis Kit (+gDNA wiper) (Cat: R412, Vazyme, China). Primers targeting the VP1 gene (GenBank ID: OQ718383.1) were designed as follows: Forward primer: 5′‐gagggaaggatttcacatatgATGTGCTCAACCTGCGCTAAC‐3′; reverse primer: 5′‐acctgcagggaattcggatccTTATACCGCTCCTAATATTTGAGGC‐3′. The VP1 gene fragment was amplified using PrimeSTAR Max DNA Polymerase Ver.2 (Cat: RO47S, Takara, China) and verified by 1% agarose gel electrophoresis. The pMAL‐C5x(+) vector was double‐digested with *NdeI* and *EcoRI* (Cat: R0111, R3101, NEB, USA). Both the PCR product and linearized vector were gel‐purified (Cat: D2500, OMEGA, USA) and assembled using DNA Assembly Mix Ultra (Cat: EG15109S, Yugong Biotech, China) to generate the recombinant plasmid pMAL‐C5x‐VP1. After transformation into *E. coli* BL21(DE3)‐competent cells, transformants were selected on LB agar plates supplemented with ampicillin and incubated overnight at 37°C. A single colony was picked for colony PCR and DNA sequencing to confirm the correct construct. For protein expression, a positive clone was inoculated (1:100) into LB medium containing ampicillin and grown at 37°C with shaking until OD_600 nm_ reached 0.5–0.6. Protein expression was induced with 1 mM isopropyl β‐D‐1‐thiogalactopyranoside (IPTG) for 5 h at 37°C, 200 rpm. Cells were harvested by centrifugation, resuspended in lysis buffer (50 mM Tris‐HCl, 100 mM NaCl, 5% glycerol, pH 8.0) containing 0.5 mM phenylmethylsulfonyl fluoride, and lysed by sonication. The supernatant obtained after centrifugation was loaded onto a Dextrin Beads 6FF gravity column (Cat: SA026005, Smart‐Lifesciences, China) to purify the MBP‐tagged VP1 fusion protein. The MBP tag was subsequently cleaved using Factor Xa protease (Cat: P8010V, NEB, USA). Protein purity and identity were confirmed by SDS‐PAGE and Western blot analysis.

### 2.4. Screening of MAb and Evaluation of Their Blocking Effects

The VP1 protein was emulsified with MONTANIDE ISA 201 VG adjuvant (Cat: ISA201VG, CepiCo (Shanghai) Special Chemicals Co., Ltd., China) at a 1:1 (v/v) ratio. Mice were immunized intramuscularly three times at 14‐day intervals, with each dose containing 50 µg of VP1 protein per mouse. Serum samples were collected after the third immunization, and antibody titers were assessed by indirect ELISA (using purified VP1 as the coating antigen) and indirect immunofluorescence assay (IFA). Mice showing satisfactory serum titers were administered a final intraperitoneal booster injection of 50 µg protein 3–5 days before cell fusion. Splenocytes harvested from immunized mice were fused with SP2/0 myeloma cells following established protocols [25]. Approximately 10 days postfusion, the supernatant from wells containing hybridoma clusters was screened for antigen‐specific antibodies using IFA. Subsequently, the cells in the positive wells were aspirated and transferred to flow cytometry tubes. A professional then performed a single‐cell isolation operation using flow cytometry. Following expansion of sorted clones, culture supernatants from positive hybridomas were collected for further characterization. To evaluate the blocking activity of each mAb, VP1 protein was coated onto ELISA plates. Wells were incubated with either positive or negative FCV reference sera as primary antibodies, followed by the respective mAb supernatant and goat anti‐mouse IgG‐horseradish peroxidase (HRP) as secondary and tertiary antibodies, respectively. Absorbance at OD_450 nm_ was measured, and the blocking rate was calculated for each mAb. The clone exhibiting the strongest blocking effect was selected for subsequent experiments.

### 2.5. MAb Purification and Labeling

Female BALB/c mice aged 7–8 weeks were used for ascites production. Mice were first primed by an intraperitoneal injection of 0.5 mL mineral oil (Cat: ST275, Beyotime, China). After 3–5 days, ~1.5 × 10^6^ hybridoma cells were injected intraperitoneally into each mouse. Ascites fluid was collected 5–7 days postinoculation, clarified by centrifugation (10,000 rpm, 10 min, 4°C), and the supernatant stored at −80°C for subsequent use. MAbs were purified from ascites using an rProtein A Beads gravity column (Cat: SA012005, Smart‐Lifesciences, China) following the manufacturer’s instructions. To determine mAb titer, an indirect ELISA was performed: purified VP1 protein was coated as the capture antigen; serially diluted mAb (starting at 1:200, with two‐fold dilutions) served as the primary antibody; ascites from SP2/0‐inoculated mice was used as the negative control; and goat anti‐mouse IgG‐HRP was employed as the secondary antibody. Finally, the purified mAbs were conjugated with HRP using a commercial labeling kit (Cat: ab102890, Abcam, UK), according to the manufacturer’s protocol.

### 2.6. IFA

Feline kidney cells were seeded in a 96‐well plate. When cells reached ~80% confluency, the culture medium was removed and replaced with the FCV inoculum. The plate was incubated at 37°C under 5% CO_2_ for 4 h until the cytopathic effect (CPE) became visible. After discarding the viral supernatant, cells were gently washed three times with PBS and fixed with 100 µL ice‐cold absolute ethanol per well for 30 min at 4°C. Following fixation, ethanol was removed, and cells were washed three times with PBS. Each well was then incubated with 50 µL hybridoma cell culture supernatant at 37°C for 60 min, followed by three washes with PBST. Subsequently, 50 µL of FITC‐conjugated goat anti‐mouse IgG diluted in PBS was added to each well, and the plates were incubated at 37°C for 60 min in the dark. After three final PBST washes, the cell nuclei were counterstained with DAPI. Fluorescence signals were visualized and recorded using a fluorescence microscope.

### 2.7. Western Blot Analysis

Recombinant VP1 protein was mixed with 6× SDS‐PAGE loading buffer and boiled at 100°C for 10 min, and 10 µL of the denatured sample was loaded per well. Electrophoresis was performed on a polyacrylamide gel at 80 V for 30 min, followed by 120 V for 60 min. Separated proteins were transferred onto a polyvinylidene difluoride membrane using standard wet‐transfer conditions. The membrane was blocked with 5% skim milk in PBST at room temperature for 1 h, followed by three washes with PBST (10 min each). The membrane was then incubated with the mAb (primary antibody) diluted in blocking solution for 1 h at room temperature and washed three times with PBST. Subsequently, the membrane was incubated with goat anti‐mouse IgG (H + L)‐HRP conjugate (secondary antibody) in the dark for 1 h at room temperature, followed by three final washes with PBST. Protein bands were visualized using a chemiluminescence imaging system.

### 2.8. Establishment and Optimization of a Blocking ELISA

Optimal assay conditions were determined using a checkerboard titration design. Purified VP1 protein was diluted in carbonate‐bicarbonate buffer (pH 9.6) to concentrations ranging from 0.5 to 5 μg/mL, and 100 μL of each dilution was coated onto 96‐well ELISA plates by overnight incubation at 4°C. Plates were then washed three times with PBST. For blocking, various blocking buffers were evaluated, including 1% BSA, 1%–5% skim milk, and 1% gelatin in PBST. Blocking was performed at 37°C for durations between 30 and 150 min, followed by three washes with PBST. Positive and negative FCV reference sera were serially diluted (from 1:2 to 1:64), added at 100 μL/well, and incubated at 37°C for 15–150 min. After washing, the HRP‐labeled mAb was applied at dilutions of 1:100 to 1:3200 (100 μL/well) and incubated at 37°C for 15–150 min. Color development was initiated by adding 100 μL TMB substrate per well and incubating at room temperature for 5–30 min. The reaction was stopped with 50 μL of 2 M sulfuric acid per well. Absorbance at 450 nm was measured for both positive (P) and negative (N) controls. The condition yielding the lowest P/N ratio was selected as optimal for subsequent assays.

### 2.9. Determination of the Cutoff Value and Evaluation of Specificity, Sensitivity, and Repeatability

A panel of 86 FCV‐negative and 78 FCV‐positive serum samples, previously confirmed by IFA, were analyzed using the optimized blocking ELISA. The sample‐to‐negative (P/N) ratio and percent inhibition (PI) values were calculated for each sample. Receiver operating characteristic (ROC) curve analysis was performed using GraphPad Prism 8 and SPSS Statistics 17.0 to determine the assay cutoff. The Youden index was derived from specificity and sensitivity data, and the PI value corresponding to the maximum Youden index was selected as the diagnostic cutoff. To evaluate specificity, the assay was applied to known positive sera for other common feline pathogens, including feline herpesvirus type I, feline panleukopenia virus, *Salmonella*, *Escherichia coli*, and *Mycoplasma*, using FCV‐positive and FCV‐negative sera as controls. To assess analytical sensitivity, serial dilutions (1:2 to 1:1024) of FCV strongly positive, positive, weakly positive, and negative sera were tested to determine the highest detectable dilution. For repeatability testing, three independent batches of ELISA plates were prepared (10 plates per batch). Five plates from each batch were randomly selected to test the same panel of FCV sera (strongly positive, positive, weakly positive, and negative) in intra‐ and inter‐batch runs. Coefficients of variation (CV) were calculated to evaluate assay reproducibility.

### 2.10. Clinical Sample Analysis

A total of 105 field feline serum samples (the cat serum samples we collected mainly included those from cats that had received the cat trivalent vaccine, healthy cats, cats suspected of being infected with the FCV virus, and cats with respiratory symptoms) were tested in parallel using the optimized blocking ELISA established in this study and an indirect ELISA method based on the protocol described in DB22/T 3035‐2019 detection of conventional experimental cat FCV ELISA method. Results obtained from both assays were compared, and the overall agreement rate between the two methods was calculated.

### 2.11. Statistical Analysis

Statistical analyses were performed using GraphPad Prism 8 (San Diego, CA, USA) and SPSS Statistics 17.0. The cutoff value for the blocking ELISA was determined based on sensitivity, specificity, Youden’s index, and the area under the ROC curve (AUC). The repeatability of the assay was assessed using the mean, standard deviation, and CV. The agreement between the blocking ELISA and a commercial indirect ELISA kit was evaluated with Cohen’s kappa coefficient.

## 3. Results and Analysis

### 3.1. Protein Expression and Characterization

**Figure 1:**
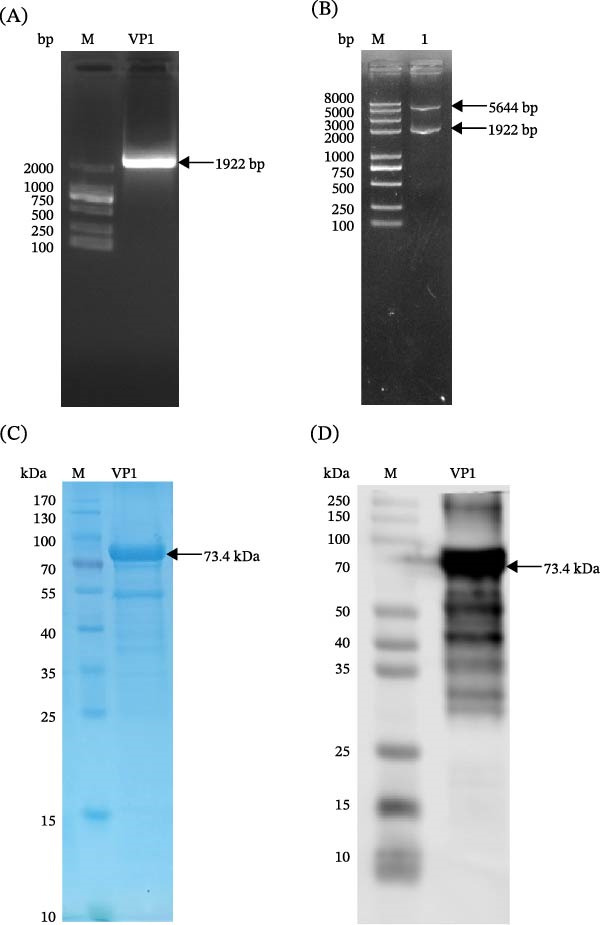
Construction of the recombinant pMAL‐C5x‐VP1 vector and purification and identification of the VP1 protein. (A) PCR amplification of the FCV VP1 fragment. (B) Double digestion of the recombinant pMAL‐C5x‐VP1 vector with *NdeI* and *EcoRI*. (C) SDS‐PAGE analysis of the purified recombinant VP1 protein. (D) Western blot analysis of the recombinant VP1 protein probed with FCV‐positive serum.

To obtain both the immunogen and coating antigen, the VP1 protein of FCV was expressed using a prokaryotic system. Amplification of the VP1 gene from FCV cDNA yielded a product of ~1992 bp, as confirmed by 1% agarose gel electrophoresis (Figure 1A), consistent with the expected size. The amplified fragment was cloned into the pMAL‐C5x(+) expression vector. Double digestion with *NdeI* and *EcoRI* released both the vector backbone and the insert fragment (Figure 1B), confirming successful plasmid construction. Following transformation, expression induction, and purification, SDS‐PAGE analysis revealed a recombinant protein with a molecular weight of ~73.4 kDa and a purity of about 90% (Figure 1C). Western blot analysis using FCV‐positive serum detected a specific band at the same molecular weight (Figure 1D), indicating that the purified recombinant VP1 protein retained its antigenicity.

### 3.2. Generation and Characterization of MAb

**Figure 2:**
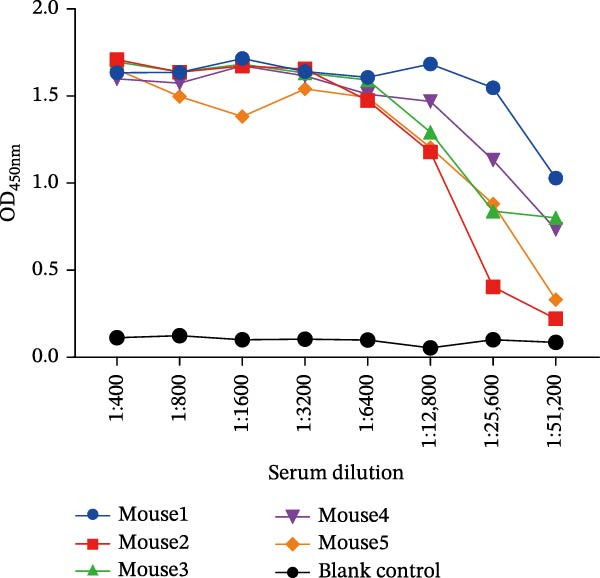
Serum antibody titers in mice after three immunizations with FCV VP1 protein, measured by indirect ELISA. Mouse 1–5: immunized with recombinant VP1 protein; blank control: no immunization.

**Figure 3:**
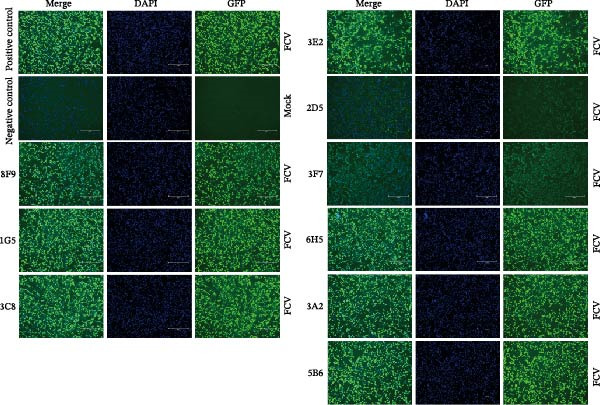
Detection of mAb reactivity by IFA. Positive control: serum from VP1‐immunized mice; negative control: DMEM medium; others: supernatants from nine monoclonal hybridoma cell lines.

**Figure 4:**
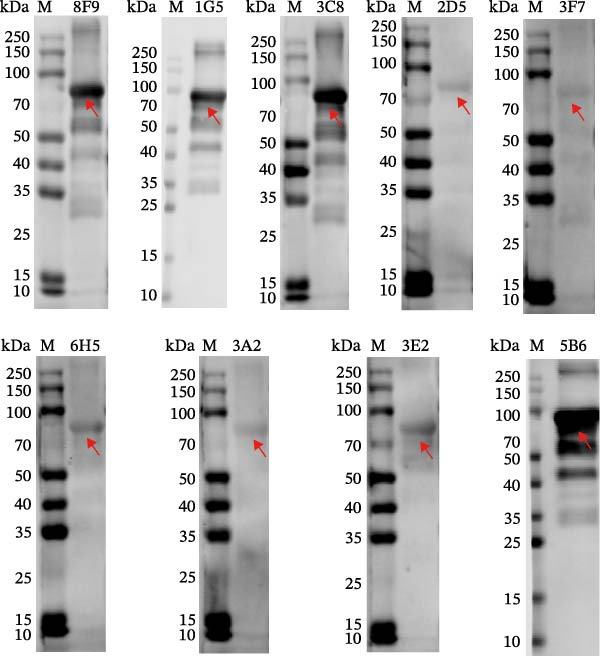
Western blot analysis of nine mAbs against recombinant FCV VP1 protein.

**Figure 5:**
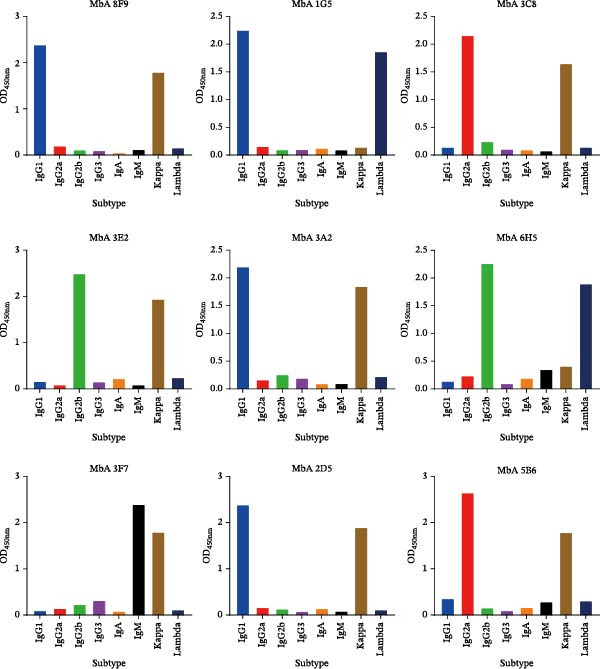
Subclass analysis of nine mAbs using a commercial antibody isotyping kit.

**Figure 6:**
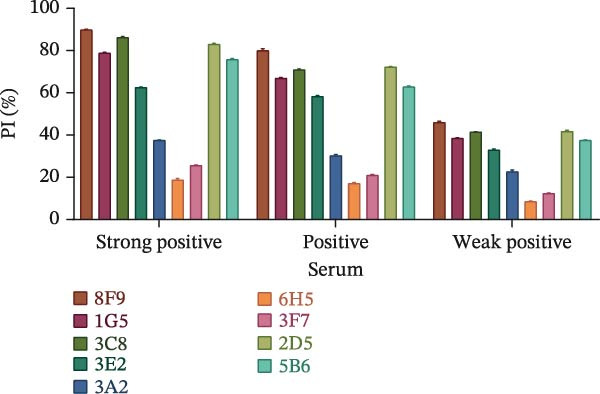
Blocking activity of nine mAbs against FCV strongly positive, positive, and weakly positive serum samples.

To evaluate the immunogenicity of the recombinant VP1 protein, mouse serum was collected 14 days after the third immunization and analyzed by indirect ELISA and IFA. The results indicated serum titers exceeding 1:51,200 by indirect ELISA (Figure 2) and 1:1600 by IFA, confirming strong immunogenicity suitable for hybridoma generation. Splenocytes from immunized mice were fused with SP2/0 myeloma cells. Screening by IFA identified nine hybridoma cell lines that stably secreted mAbs against FCV VP1 (Figure 3). Western blot analysis using the nine mAb supernatants as primary antibodies demonstrated that all recognized the denatured recombinant VP1 protein, producing distinct bands (Figure 4). Together, the Western blot and IFA results indicate that the selected mAbs recognize both linear and conformational epitopes. Subclass analysis using a commercial mAb isotyping kit (Cat: ab273149, Abcam, UK) revealed that three mAbs were IgG1/κ, two were IgG2a/κ, one each was IgG1/λ, IgG2b/κ, and IgG2b/λ, and one was IgM/κ (Figure 5). The blocking activity of each mAb was assessed in a competitive ELISA format: recombinant VP1 was coated as the antigen, FCV‐positive serum served as the primary antibody, the respective mAb as the secondary antibody, and goat anti‐mouse IgG‐HRP as the tertiary antibody. Seven of the nine mAbs exhibited blocking activity, with mAb 8F9 showing the highest blocking rate (Figure 6). Therefore, mAb 8F9 was selected for subsequent assay development.

### 3.3. Purification and HRP Conjugation of mAbs

**Figure 7:**
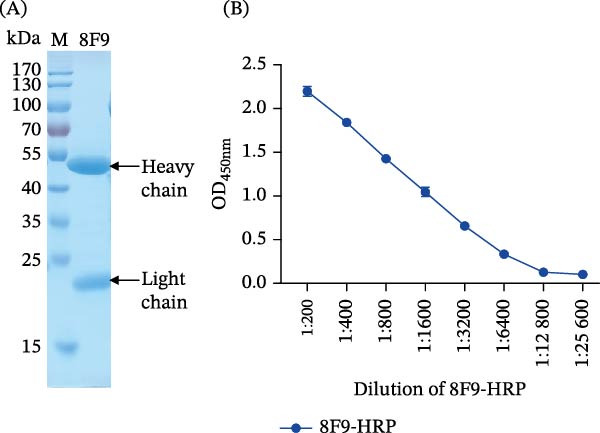
Purification and HRP‐labeling analysis of mAb 8F9. (A) SDS‐PAGE of antibody purified by rProtein G Beads gravity column. (B) Direct ELISA analysis of HRP‐conjugated 8F9 mAb titer.

To scale up antibody production, female BALB/c mice (7–8 weeks old) were first primed by an intraperitoneal injection of mineral oil. One week later, the 8F9 hybridoma cell line was injected intraperitoneally. Ascites fluid was harvested 5–7 days postinoculation and purified using an rProtein G Beads gravity column, followed by conjugation to HRP. Under denaturing conditions, SDS‐PAGE analysis of the purified antibody revealed high purity, showing clear bands corresponding to the heavy chain (~55 kDa) and light chain (~25 kDa) (Figure 7A). The titer of the HRP‐conjugated mAb was determined by direct ELISA, reaching 1:6400 (Figure 7B).

### 3.4. Optimization of Blocking ELISA Reaction Conditions

**Figure 8:**
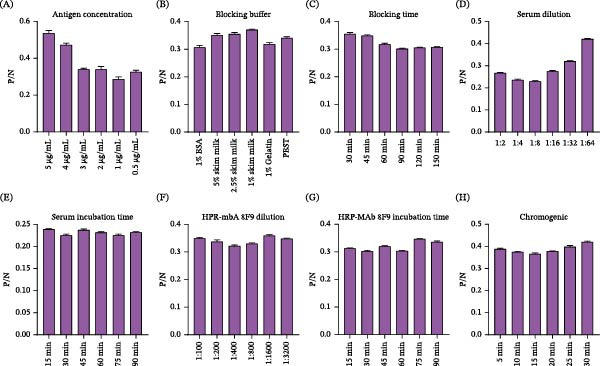
Results of optimization of reaction conditions for blocking ELISA method. (A) The optimal antigen coating concentration is 1 μg/mL; (B) the optimal blocking agent is 1% BSA; (C) the optimal blocking time is 90 min; (D) the optimal dilution ratio of the test serum is 1:8; (E) the optimal incubation time of serum is 30 min; (F) the optimal dilution ratio of HRP‐mAb 8F9 is 1:400; (G) the optimal action time of HRP‐mAb 8F9 is 30 min; and (H) the optimal color development time is 15 min.

To enhance the performance of the blocking ELISA, key reaction parameters were systematically optimized. The following conditions were selected based on achieving the lowest positive‐to‐negative (P/N) ratio: coating antigen concentration: 1.0 μg/mL recombinant VP1 (Figure 8A); blocking reagent: 1% BSA (Figure 8B); blocking time: 90 min (Figure 8C); serum dilution: 1:8 (Figure 8D); serum incubation: 30 min at 37°C (Figure 8E); HRP‐conjugated 8F9 MAb dilution: 1:400 (Figure 8F); MAb incubation: 30 min at 37°C (Figure 8G); TMB substrate incubation: 15 min at room temperature (Figure 8H).

### 3.5. Determination of the Diagnostic Cutoff for the Blocking ELISA

**Figure 9:**
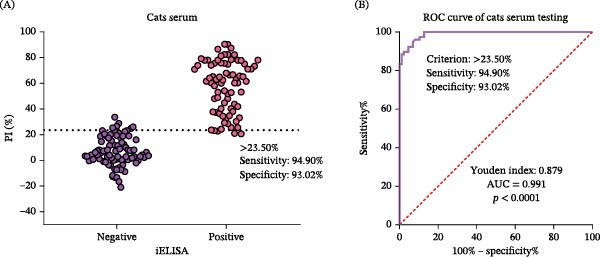
Detection of feline serum samples and ROC analysis. (A) Blocking ELISA results from 86 FCV‐negative and 78 FCV‐positive serum samples. (B) ROC curve analysis of the 164 serum samples.

Using the optimized conditions, the blocking ELISA was applied to 86 FCV‐negative and 78 FCV‐positive serum samples. The diagnostic performance was evaluated by ROC curve analysis (SPSS software). The optimal cutoff for PI was determined to be 23.5%, yielding a sensitivity of 94.9% and a specificity of 93.02%, with a corresponding Youden index of 0.879. The AUC was 0.991 (95% CI: 0.9817–0.9994, *p* < 0.0001) (Figure 9A, B). Thus, under valid assay conditions, serum samples with a PI value <23.5% were classified as FCV‐antibody negative, while those with a PI value ≥23.5% were classified as FCV‐antibody positive.

### 3.6. Evaluation of the Blocking ELISA Performance

**Figure 10:**
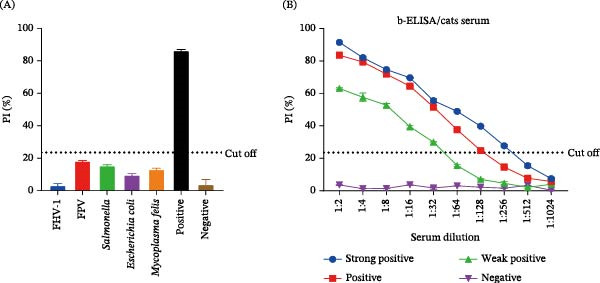
Evaluation of the specificity and analytical sensitivity of the blocking ELISA. (A) Assessment of cross‐reactivity with positive sera against other common feline pathogens. (B) Determination of the maximum detectable dilution for FCV‐strongly positive, FCV‐positive, and FCV‐weakly positive sera.

To evaluate the specificity of the established blocking ELISA, sera positive for other common feline pathogens—feline herpesvirus type I, feline panleukopenia virus, *Salmonella*, *Escherichia coli*, and feline *Mycoplasma—*were tested under the optimized assay conditions. FCV‐positive and FCV‐negative sera served as controls. The assay specifically detected FCV antibodies and showed no cross‐reactivity with the other five pathogen‐positive sera (Figure 10A), confirming its high specificity. Analytical sensitivity was assessed by testing serial two‐fold dilutions of FCV‐strongly positive, FCV‐positive, FCV‐weakly positive, and FCV‐negative sera. The highest detectable dilutions were 1:256 for strongly positive, 1:128 for positive, and 1:32 for weakly positive sera, while the negative serum showed no reactivity (Figure 10B). To evaluate the assay reproducibility, three independent batches of ELISA plates were prepared. Five plates from each batch were randomly selected to test the same panel of FCV sera (strongly positive, positive, weakly positive, and negative) in intra‐ and inter‐batch runs. The CV for intra‐batch tests were 0.96%, 0.83%, 2.88%, and 5.38%, respectively; for inter‐batch tests, the CVs were 0.79%, 0.71%, 3.14%, and 7.05%, respectively (Table 1). All CV values were below 10%, indicating good repeatability and stability of the established blocking ELISA.

**Table 1:** Reproducibility verification of the blocking ELISA method.

Samples	Intra‐coefficient of variation		Inter‐coefficient of variation	
	Mean PI (%) ± SD	CV (%)	Mean PI (%) ± SD	CV (%)
Strong positive	90.58 ± 0.87	0.96	90.66 ± 0.79	0.87
Positive	79.53 ± 0.66	0.83	80.04 ± 0.71	0.89
Weak positive	36.46 ± 1.05	2.88	36.34 ± 1.14	3.14
Negative1	8.546 ± 0.46	5.38	8.223 ± 0.58	7.05

### 3.7. Clinical Application

To evaluate the clinical applicability of the established blocking ELISA, a panel of 105 field feline serum samples was tested in parallel using the optimized blocking ELISA and the indirect ELISA antibody detection method described in DB22/T 3035–2019 (ELISA method for detection of FCV antibodies in laboratory cats). The agreement between the two assays was assessed. The results showed that the seropositive rate of FCV in the tested population was 24.76% (26/105). The overall agreement between the blocking ELISA and the reference indirect ELISA was 96.55% (Table 2), with a Cohen’s kappa value of 0.9, indicating excellent concordance. These findings demonstrate that the blocking ELISA developed in this study is a reliable tool for FCV antibody detection and vaccine immune response evaluation.

**Table 2:** Comparison of clinical sample cat serum testing and indirect ELISA antibody detection methods^a^.

Blocking ELISA	Indirect ELISA			
	Positive	Negative	Aggregate	Conformity rate (%)
Positive	23	3	26	88.46
Negative	1	78	79	98.73
Aggregate	24	81	105	96.19

^a^Kappa = 0.9.

## 4. Discussion

With the notable increase in cat‐owning households and the widespread use of the feline trivalent vaccine (targeting feline panleukopenia virus, feline herpesvirus, and FCV), research interest in FCV has grown considerably [26]. FCV is now endemic not only in domestic cat populations worldwide but also in various wild feline species, including tigers and lions. Young cats are the most susceptible, exhibiting significantly higher morbidity than adults. Infected cats may shed the virus persistently, and the virus can remain infectious in the environment for at least 28 days. The RNA genome of FCV is prone to genetic variation, which limits the cross‐protective efficacy of current vaccines against certain circulating strains. As the VP1 capsid protein constitutes the primary immunogen of FCV, there is an urgent need to elucidate its immune protection domains and neutralizing epitopes and to develop specific, sensitive diagnostic methods for FCV detection [27, 28].

In terms of serological detection, Ji et al. [29] successfully expressed the recombinant VP1 protein of FCV in 2025 and developed an indirect ELISA for detecting FCV antibodies. This assay exhibited high performance, showing no cross‐reactivity with FPV‐ or FHV‐positive sera and achieving a minimum detectable titer of 1:10,240 for FCV‐positive sera. The intra‐ and inter‐batch CV were below 10%, and the concordance rate with commercial kits reached 100%. While indirect ELISA is simple, cost‐effective, and versatile, its specificity can be compromised by nonspecific antibodies. In contrast, blocking ELISA operates on a competitive inhibition principle, wherein antibodies in the test sample compete with a known standardized antibody for binding to the immobilized antigen. The resulting signal inhibition rate is directly proportional to the antibody concentration, conferring high specificity. In this study, a blocking ELISA based on a mAb targeting the FCV VP1 protein was developed. Through systematic optimization, the following optimal reaction conditions were established: a coating antigen concentration of 1.0 μg/mL, blocking with 1% BSA, and a test serum dilution of 1:8, among others. ROC curve analysis determined the cutoff PI value to be 23.5%, yielding a sensitivity of 94.9% and a specificity of 93.02%. This critical value was derived based on a limited sample (86 negative sera and 78 positive sera) under laboratory conditions. However, when it is applied in future clinical settings and expanded to larger‐scale and more diverse field populations, this critical value still requires further verification and may need to be appropriately adjusted according to the actual situation. The method demonstrated excellent specificity, with no cross‐reactivity against common feline pathogens, and high analytical sensitivity, enabling detection of strongly positive sera at dilutions up to 1:256. Repeatability was robust, with intra‐ and inter‐batch CV below 10%. Clinical evaluation revealed a total concordance rate of 96.19% with the indirect ELISA reference method recommended in the local standard DB22/T 3035–2019, with a Cohen’s kappa value of 0.9, indicating near‐perfect agreement. These findings confirm that the established blocking ELISA is a reliable and practical tool for FCV antibody detection and postvaccination immune evaluation.

The VP1 protein of FCV comprises two key structural domains: the internal shell (S) domain, which forms the core architecture of the viral capsid, and the exposed protrusion (P) domain, which is further subdivided into the P1 and P2 subdomains [30]. The P2 subdomain, located on the outermost surface, plays a central role in both infection and host immunity—it serves as the primary interface for virus attachment to host cell receptors and is a critical target for host immune recognition [31, 32]. Although FCV exists as a single serotype, the VP1 protein, particularly the hypervariable P2 subdomain, exhibits substantial genetic diversity. This variability restricts cross‐protective immunity among different strains and elevates the risk of vaccine breakthrough [33, 34]. Among the six defined antigenic regions (A–F) of VP1 [9, 10], the E region—situated within the P2 subdomain—is of particular importance. It harbors multiple immunogenic epitopes capable of eliciting neutralizing antibodies and constitutes the core region responsible for mediating effective humoral immunity [11]. Consequently, precise characterization of the E region and its constituent epitopes is essential not only for understanding FCV immune evasion strategies but also for guiding the development of improved diagnostic tools and broadly protective vaccines [13, 35]. In this study, the FCV VP1 protein was expressed at high yield and purity using an *E. coli* prokaryotic expression system, thereby minimizing nonspecific interference associated with crude whole‐virus antigens. Following three rounds of immunization, indirect ELISA demonstrated that mouse serum titers exceeded 1:51,200 (Figure 2), and indirect IFA confirmed titers greater than 1:1600, indicating strong immunogenicity of the recombinant VP1 protein. Using VP1‐immunized mice, nine hybridoma cell lines secreting mAbs with blocking activity were generated via cell fusion technology. Ascites fluid was produced by intraperitoneal inoculation of BALB/c mice, and mAbs were purified to high purity using rProtein G Beads affinity chromatography. These mAbs, employed as competitive antibodies in conjunction with purified VP1 protein as the coating antigen, specifically recognized distinct epitopes within VP1 and effectively blocked the nonspecific binding of irrelevant antibodies present in test sera. This strategy substantially enhanced the specificity and accuracy of the resulting immunoassay.

Despite the advantages of the VP1 protein‐based blocking ELISA—including high specificity and operational standardization—its application in FCV antibody detection still faces certain limitations. Given the considerable sequence variability of the VP1 protein, particularly within the hypervariable P2 subdomain, mAbs that target a single epitope may exhibit reduced detection sensitivity due to epitope divergence among circulating strains, potentially leading to false‐negative results. To address this, future work will focus on the fine epitope mapping of the selected mAbs to assess the conservation of their target epitopes across diverse FCV field isolates. In parallel, we plan to validate the analytical performance of the established method using a panel of feline serum samples derived from infections with phylogenetically distinct FCV strains. These efforts will provide critical insights into the broad‐spectrum reactivity and practical robustness of the assay, thereby laying the foundation for the development of a more universally applicable FCV antibody detection system. Furthermore, although we have confirmed the diagnostic potential of the selected mAbs, our cell‐level validation revealed that these mAbs do not possess FCV‐neutralizing activity. Therefore, their application is limited to the diagnostic field rather than for treatment or passive immunity. Future research can attempt to obtain antibodies with neutralizing activity through antibody engineering modification or targeted screening of neutralizing epitopes for more prevalent FCV strains.

## 5. Conclusion

In this study, nine mAbs capable of recognizing both linear and conformational epitopes of the FCV VP1 protein were successfully generated. Among them, mAb 8F9 (isotype IgG2a/κ) exhibited the strongest blocking activity and was selected to establish a blocking ELISA for the detection of FCV antibodies. The developed assay is operationally simple, yields stable and reproducible results, and is well suited for large‐scale FCV antibody screening, postvaccination immune response evaluation, and antibody surveillance in SPF cat colonies. Collectively, this method provides robust technical support for the control of FCV‐associated diseases and the health management of feline populations.

## Author Contributions

Conceptualization, funding acquisition, resources, supervision: Shuyan Wu, Changyou Xia, and He Zhang. Data curation, investigation, methodology: Haojie Wang,Lihong Xue, Bowen Shan, Shuyan Wu, and Tongqin An. Formal analysis: Haojie Wang, Lihong Xue, and Bowen Shan. Validation, writing – original draft, review and editing: Haojie Wang, Lihong Xue, Changqing Yu, Changyou Xia, and He Zhang.

## Funding

The research was supported by grants from the National Natural Science Foundation of China‐Youth Science Fund (Grant 32500449), the Heilongjiang Province Natural Fund Joint Guidance Project (Grant LH2024C059), the National Key R&D Program of China (Grants 2023YFF0724603 and 2023YFF0724604), and the Prevention and Control of Emerging and Major Infectious Diseases‐National Science and Technology Major Project (Grant 2025ZD01900703).

## Conflicts of Interest

The authors declare no conflicts of interest.

## Data Availability

The data that support the findings of this study are available from the corresponding author upon reasonable request. The data that support the findings of this study are not publicly available due to restrictions concerning the source of the animal sera.
